# HLA-G Expression on Blasts and Tolerogenic Cells in Patients Affected by Acute Myeloid Leukemia

**DOI:** 10.1155/2014/636292

**Published:** 2014-03-13

**Authors:** Grazia Locafaro, Giada Amodio, Daniela Tomasoni, Cristina Tresoldi, Fabio Ciceri, Silvia Gregori

**Affiliations:** ^1^San Raffaele Telethon Institute for Gene Therapy (HSR-TIGET), Division of Regenerative Medicine, Stem Cells and Gene Therapy, San Raffaele Scientific Institute (HSR-DREaM-GENE), Via Olgettina 58, 20132 Milan, Italy; ^2^Ph.D Program in Translational and Molecular Medicine (DIMET), University of Milan-Bicocca, Piazza della Scienza 3, 20126 Milan, Italy; ^3^Immunohematology Blood Transfusion Service (S.I.M.T.), San Raffaele Hospital, Via Olgettina 60, 20132 Milan, Italy; ^4^Hematology and Bone Marrow Transplantation Unit, Division of Regenerative Medicine, Stem Cells and Gene Therapy, San Raffaele Scientific Institute (HSR-DREaM-GENE), Via Olgettina 58, 20132 Milan, Italy

## Abstract

Human Leukocyte Antigen-G (HLA-G) contributes to cancer cell immune escape from host antitumor responses. The clinical relevance of HLA-G in several malignancies has been reported. However, the role of HLA-G expression and functions in Acute Myeloid Leukemia (AML) is still controversial. Our group identified a subset of tolerogenic dendritic cells, DC-10 that express HLA-G and secrete IL-10. DC-10 are present in the peripheral blood and are essential in promoting and maintaining tolerance via the induction of adaptive T regulatory (Treg) cells. We investigated HLA-G expression on blasts and the presence of HLA-G-expressing DC-10 and CD4^+^ T cells in the peripheral blood of AML patients at diagnosis. Moreover, we explored the possible influence of the 3′ untranslated region (3′UTR) of *HLA-G*, which has been associated with HLA-G expression, on AML susceptibility. Results showed that HLA-G-expressing DC-10 and CD4^+^ T cells are highly represented in AML patients with HLA-G positive blasts. None of the HLA-G variation sites evaluated was associated with AML susceptibility. This is the first report describing HLA-G-expressing DC-10 and CD4^+^ T cells in AML patients, suggesting that they may represent a strategy by which leukemic cells escape the host's immune system. Further studies on larger populations are required to verify our findings.

## 1. Introduction

Human Leukocyte Antigen (HLA)-G is a nonclassical HLA class I molecule, originally described essential for promoting fetus-maternal tolerance [1, 2]. It is now clear that HLA-G is involved in promoting beneficial tolerance in several settings, such as autoimmunity and organ transplantation, and in contributing to detrimental tolerance in viral infections and cancer [3]. HLA-G is expressed in seven different isoforms, four of which are membrane-bound (HLA-G1, G2, G3, and G4) and three are soluble (HLA-G5, G6, and G7) [4, 5]. Among these isoforms, the best characterized are HLA-G1, the most stable membrane-bound isoform, soluble HLA-G5, and shed HLA-G1. HLA-G modulates immune responses through several nonexclusive mechanisms: it inhibits cytolytic activities of NK and CD8^+^ cytotoxic T cells [6] and proliferation of T cells [7, 8], and it modulates antigen presenting cell (APC) differentiation and function [9]. APCs overexpressing HLA-G are poor stimulators and are able to induce anergic/suppressor CD4^+^ T cells [9–12]. Our group described a subset of tolerogenic IL-10-producing DC (DC-10) that is present in the peripheral blood [13, 14]. DC-10 are characterized by the expression of membrane-bound HLA-G and by their ability to induce adaptive IL-10-producing T regulatory (Treg) cells [13, 14]. We demonstrated that DC-10 accumulate in human decidua during pregnancy where they contribute in generating a tolerogenic microenvironment limiting immune responses* in vivo* [15].

Studies on solid tumors revealed that HLA-G could be potentially expressed by all tumors, either as membrane-bound or as soluble isoform [16, 17]. In these contexts, HLA-G acts as a negative regulator of the immune response through different mechanisms, including inhibition of angiogenesis, prevention of antigen recognition and T cell migration, and suppression of T and NK cytotoxicity [16, 18, 19]. HLA-G expression by leukemic cells is still controversial. Analysis performed on blasts from patients with different leukemia, including Acute Myeloid Leukemia (AML), Acute Lymphoid Leukemia (ALL), and Chronic Myeloid Leukemia (CML), demonstrated that neither mRNA for any HLA-G isoforms nor HLA-G antigen was detected [20, 21]. However, more recently it has been shown that the expression of HLA-G by circulating blasts from AML, CML, but not B-ALL, and by B-CLL cells was strongly associated with an unfavorable outcome of the disease [22–24]. In addition, a correlation between soluble HLA-G plasma levels and AML, ALL, and B-CLL was proposed [25, 26].

Despite low degree of genetic variability in the coding region of* HLA-G*, several polymorphisms are present in the noncoding region of the gene both at 5′ upstream regulatory region (URR) and 3′ untranslated region (UTR), which may influence the HLA-G expression [27]. The most studied polymorphism at the 3′ UTR is the presence (Ins) or absence (Del) of a fragment of 14 base pairs (14 bp Ins/Del) that has been associated with* HLA-G* mRNA stability [28–30]. In addition, the +3142 C/G single nucleotide polymorphism (SNP) controls the magnitude of mRNA production, since the presence of the G may increase the affinity of this region for miR-148a, miR-148b, and miR-152 [31, 32]. The +3187 A/G SNP has been reported to affect mRNA stability due to its proximity to an AU-rich motif, which mediates the* HLA-G* mRNA degradation [33]. Beside these polymorphic sites, other less studied SNPs of the 3′UTR are located at positions +3001 T/C, +3003 T/C, +3010 C/G, +3027 C/A, +3035 C/T, and +3196 C/G [34, 35].

The 14 bp Ins/Del has been associated with tolerance in different clinical conditions including autoimmunity [36–38], pathological pregnancy [39–42], recurrent spontaneous abortions [39, 40, 42, 43], and preeclampsia [29, 41, 43–45], although the results on the two latter conditions are contradictory. The presence of the 14 bp Del has been found to be predictive of the incidence of graft versus host disease after unrelated [46] and HLA-identical sibling [47] hematopoietic stem cell transplantation (HSCT) for beta-Thalassemia, suggesting a role for this polymorphism in the establishment of immunological tolerance also in the context of HSCT. The association of HLA-G polymorphisms with malignancies has been studied in a wide range of solid tumors, including breast and cervical cancers [48–52], but, thus far, it has not been evaluated in leukemia.

We investigated the expression of HLA-G on leukemic blasts and tolerogenic immune cells, DC-10 and CD4^+^ T cells, in the peripheral blood of AML patients at diagnosis. We also determined whether polymorphisms at 3′ UTR of* HLA-G* locus correlate with AML susceptibility.

## 2. Materials and Methods

### 2.1. Patients

All protocols were approved by the institutional review board and samples were collected under written informed consent according to the Declaration of Helsinki. 22 patients affected by AML were included in this retrospective study and analyzed for biological and clinical characteristics. AML diagnosis was based on standard cytological criteria according to the French-American-British (FAB) classification. Patients' diagnosis was subclassified by morphological and immune phenotyping. None of the patients received medical interventions before the study. Patients' characteristics are listed in Tables 1 and 2.

### 2.2. Cells Isolation and Serum Collection

Peripheral blood mononuclear cells (PBMCs) from AML patients were isolated by Ficoll density gradient centrifugation and cryopreserved in gas phase of liquid nitrogen to the time of analysis. Serum was obtained from the blood samples of AML patients by centrifugation and cryopreserved in gas phase of liquid nitrogen for ELISA test.

### 2.3. Cytogenetic Analysis

Cytological analysis was performed using standard G-band karyotyping technique. Results were described according to the International System for Human Cytogenetic Nomenclature [53].

### 2.4. Flow Cytometry Analysis

Frozen PBMCs were thawed in X-VIVO 15 medium (Lonza, Italy), supplemented with 5% pooled AB human serum (Lonza, Italy) and 100 U/mL penicillin/streptomycin (Lonza, Italy), and washed twice in Phosphate Buffered Saline (PBS) (Sigma, CA, USA) with 2% Fetal Bovine Serum (FBS) (Lonza, Italy). PBMCs were initially incubated for 10 min at room temperature with FcR blocking reagent (Miltenyi Biotech, Germany) and stained for additional 20 min at room temperature in the dark with monoclonal antibodies (mAbs) specific for the following human antigens: CD45 (BioLegend, USA), CD16 (BD Pharmigen, CA, USA), CD4 (Becton Dickinson, CA, USA), CD14 (Becton Dickinson, CA, USA), and HLA-G (MEM-G9, Exbio, Czech Republic). Cells were identified using a multiparametric approach based on the combination of mAbs. Samples were acquired using a FACS Canto II flow cytometer (Becton Dickinson, CA, USA), and data were analyzed with FCS express (De Novo Software, CA, USA). Quadrant markers were set accordingly to unstained controls. Leukemic blasts were identified as CD45^dim⁡^ according to Lacombe et al. [54].

### 2.5. Detection of Soluble HLA-Gs

Levels of shed HLA-G1 and soluble HLA-G5 were determined by enzyme-linked immunosorbent assay (ELISA), as previously described [55, 56]. To detect sHLA-G (shed HLA-G1 and HLA-G5) plates (Nunc-Immuno Plate PolySorp, Thermoscientific, Denmark) were coated with the mAb G233 (Exbio, Czech Republic), whereas to detect HLA-G5 plates were coated with the mAb 5A6G7 (Exbio, Czech Republic). sHLA-G or HLA-G5 was detected with biotinylated *β*_2_-microglobulin or W6/32 mAbs (Exbio, Czech Republic), respectively. Supernatants from HLA-G transfected LCL721.221 [57] and HeLa HLA-G5 transfected cells (kindly provided by Dr. R. Rizzo, Università di Ferrara) purified by affinity chromatography by using the W6/32 mAb were used for the generation of standard calibration curves for sHLA-G and HLA-G5, respectively. The limit of sensitivity was 0.5 ng/mL.

### 2.6. Amplification and Sequencing of 3′UTR of the HLA-G Gene

Genomic DNA was extracted from PBMCs using a commercial kit (QIAamp, QIAGEN, Italy) according to the manufacturer's instructions. Briefly, 100 ng of genomic DNA was amplified in a 25 *μ*L reaction containing 1X polymerase chain reaction (PCR) buffer (Roche, USA), 0.2 mM dNTP mix (Roche, USA), 1.5 mM MgCl_2_ (Roche, USA), 0.8 U* Taq* Polymerase (Roche, USA), and 1 *μ*M of each primer (For.: 5′ TCACCCCTCACTGTGACTGA 3′; Rev.: 5′ TTCTCATGTCTTCCATTTATTTTGTC 3′). The initial denaturation step was carried out at 95°C for 3 min, followed by 30 cycles at 93°C for 60 s, 58°C for 60 s, and 72°C for 60 s and by a final extension step at 72°C for 10 min. The amplification product was evaluated using a 2.5% agarose gel, purified using a commercial kit (Wizard SV Gel and PCR Clean-Up System, Promega, WI, USA) according to the manufacturer's instructions, and subjected to direct sequencing on both strands. All polymorphic sites observed at the 3′UTR were individually annotated and named according to previous reports [35].

### 2.7. Statistical Analysis

All results are presented as mean values ± SD. Comparison of parameters between subgroups of patients was performed using the nonparametric Mann-Whitney* U* test for continuous variable and Fisher's exact test for categorical data. Differences were regarded as significant at *P* < 0.05. The results were analyzed using GraphPad Prism 3.0 (GraphPad Software, USA).

## 3. Results and Discussion

**Figure 1:**
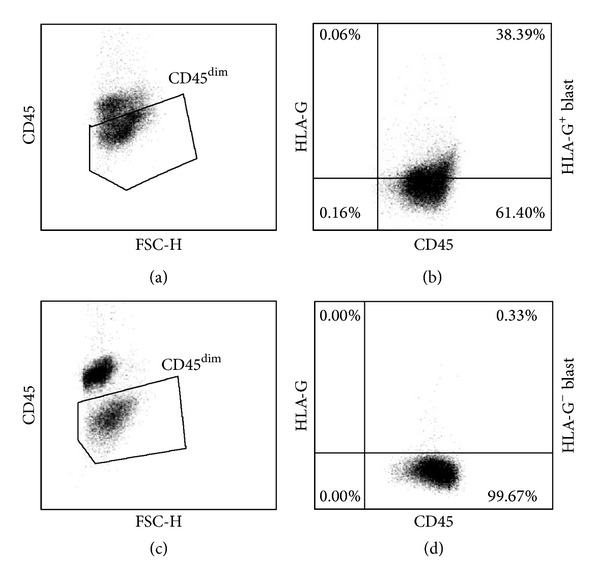
HLA-G expression on leukemic blasts. PBMCs of AML patients were analyzed by flow cytometry to determine the expression of HLA-G on blasts. An anti-human CD45 mAb was used to discriminate leukemic cells from normal mononuclear cells, being blasts CD45^dim⁡^. Representative plots from a patient with HLA-G^+^ blast ((a) and (b)) or with HLA-G^−^ blast ((c) and (d)) are shown.

We investigated the expression of HLA-G on leukemic blasts from 22 patients affected by Acute Myeloid Leukemia (AML), referring to the San Raffaele Hematology and Bone Marrow Transplantation Program (Table 1). The cohort of patients analyzed was characteristic of AML, with a median age of 59 years, both male and female (36% and 64%, resp.), with AML-M2 and AML-M4 subtype predominance. The mean percentage of blasts in the peripheral blood of patients was 65.6 ± 27.97% (mean ± SD, *n* = 22; range 13.3–96.4%). Patients' PBMCs were analyzed by flow cytometry and the expression of HLA-G was determined on blasts identified as CD45^dim⁡^ cells with MEM-G/9 mAb, which detects the full-length HLA-G1 isoform. HLA-G expression on AML blasts less than 1% was considered negative. Results showed that HLA-G was expressed in 15 out of 22 (68.2%) AML patients, and percentage of HLA-G^+^ blasts varied from 1.5% to 59% (Figure 1). Although our study has been performed in a small cohort of Caucasian AML patients, it confirmed previous analyses in Chinese AML patients [58, 59], and indicate that membrane-bound HLA-G can be expressed on AML blasts. The analysis of the association between the expression of HLA-G on blasts and clinical parameters, including patient age, gender, subtype of AML, and percentage of blasts at diagnosis, revealed that HLA-G expression is independent of all the abovementioned variables except for gender. In our cohort of AML patients, all males showed HLA-G^+^ blasts (Table 1). Nevertheless, these results are partially in accordance with previous analyses demonstrating that the HLA-G expression on leukemic blasts is not associated with specific patients' characteristics [58].

We next investigated whether HLA-G expression on blasts can be associated with cytogenetic karyotype abnormalities. Results indicated that abnormalities were present in 7 out of 15 AML patients with HLA-G^+^ blasts and only in 1 patient with HLA-G^−^ blasts (Table 2). These results are in contrast to previous data in which a marked difference in the frequency of cytogenetic abnormalities between HLA-G^+^ and HLA-G^−^ AML blasts was observed [24]. It cannot be excluded that this discrepancy can be due to the different ethnic population analyzed. Nevertheless, it remains to define whether a correlation between cytogenetic abnormalities and HLA-G expression exists by enlarging the cohort of patients.

**Figure 2:**
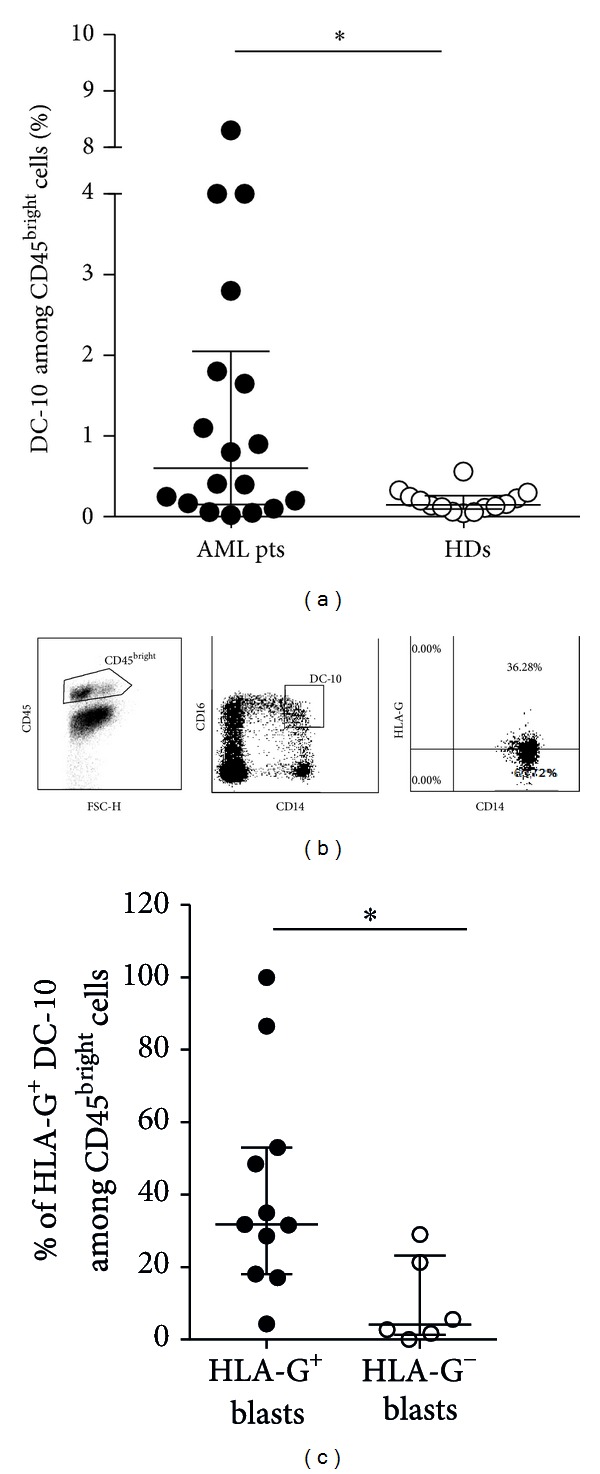
Flow cytometric analysis of cells containing DC-10 in the peripheral blood of AML patients. (a) Percentages of DC-10, identified among the CD45^bright^ cells according to the co-expression of CD14 and CD16, in the peripheral blood of AML patients (AML pts) and healthy donors (HDs) are shown. Each dot represents single AML patient or HD. Lines indicate median and interquartile range of positive cells detected in all patients and donors analyzed. *P < 0.05. (b) Representative dot plots of HLA-G^+^ DC-10 are depicted. (c) Percentages of HLA-G^+^ DC-10 in patients with HLA-G^+^ or HLA-G^−^ blasts are shown. Each dot represents single AML patient. Lines indicate the median and interquartile range of HLA-G^+^ DC-10. *P < 0.05.

**Figure 3:**
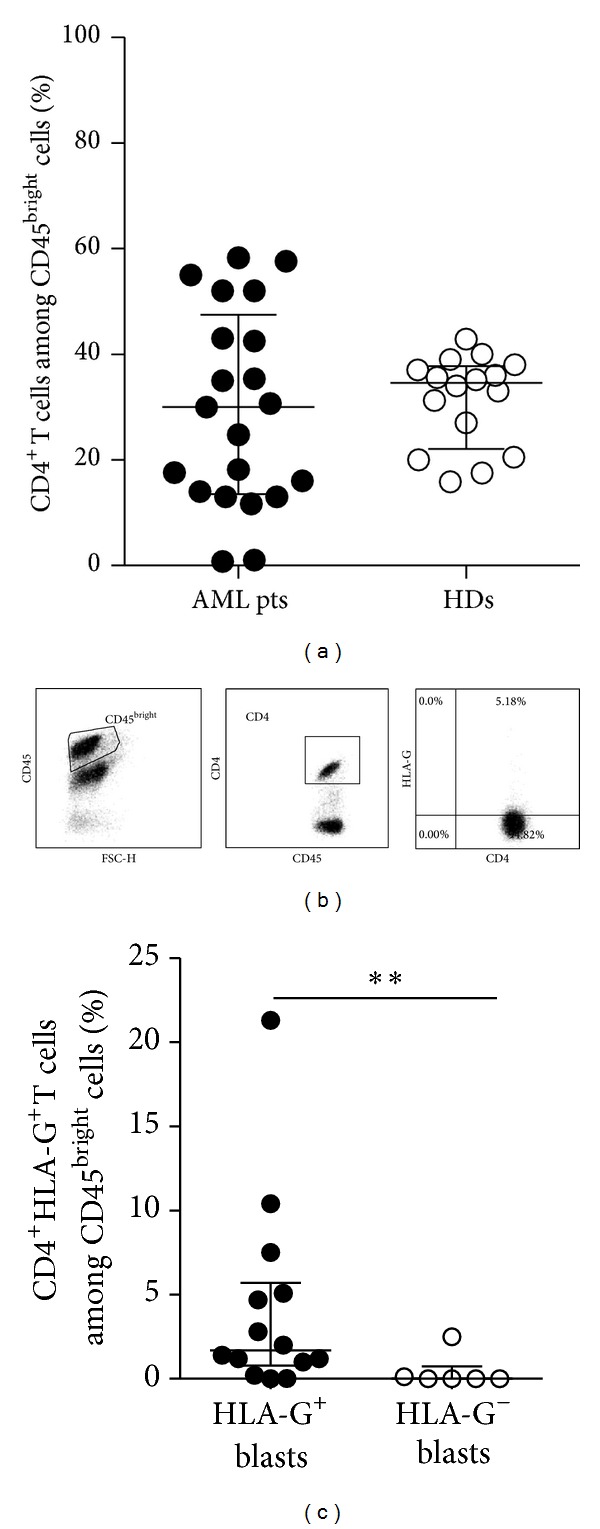
Flow cytometric analysis of CD4^+^ T cells in the peripheral blood of AML patients. (a) Percentages of CD4^+^ T cells among the CD45^bright^ cells in the peripheral blood of AML patients (AML pts) and healthy donors (HDs) are shown. Each dot represents single AML patient or HD. Lines indicate the median and interquartile range of positive cells detected in all patients and donors analyzed. (b) Representative dot plots of HLA-G^+^ CD4^+^ T cells are depicted. (c) Percentages of HLA-G^+^ CD4^+^ T cells in patients with HLA-G^+^ or HLA-G^−^ blasts are shown. Each dot represents single AML patient. Lines indicate the median and interquartile range of HLA-G^+^ CD4^+^ T cells. **P < 0.01.

One of the strategies by which tumors escape the host's immune surveillance is the upregulation of HLA-G expression on both cancer and non-tumor cells, such as mononuclear cells [17]. Our group described DC-10, which constitutively expressed HLA-G and are involved in promoting tolerance [13–15]. We postulated that DC-10 might play a role in favoring tumor escape in AML patients. The population of cells containing DC-10 was identified by the concomitant expression of CD14 and CD16 on CD45^bright^ cells. Results showed a significantly higher frequency of DC-10 in the peripheral blood of AML patients compared to that observed in healthy donors (1.5 ± 2.13%, *n* = 18, mean ± SD versus 0.19 ± 0.13%, *n* = 14, mean ± SD; *P* = 0.027; Figure 2(a)). The percentage of human DC-10 in AML patients with HLA-G^+^ and HLA-G^−^ blasts was similar (1.4 ± 2.5, *n* = 12, mean ± SD versus 1.6 ± 1.26%, *n* = 6, mean ± SD, resp.; data not shown). Previous reports indicated that HLA-G itself promotes the expression of HLA-G on T and myeloid cells [10]; thus, we compared the frequency of HLA-G-expressing DC-10 in patients with HLA-G^+^ or HLA-G^−^ blasts. Results showed a significantly higher frequency of HLA-G^+^ DC-10 in patients with HLA-G^+^ blasts as compared to those with HLA-G^−^ blasts (41.3 ± 29.25%, mean ± SD, *n* = 12 versus 10.05 ± 12.08%, mean ± SD, *n* = 6; *P* = 0.013, Figures 2(b) and 2(c)). In parallel, the presence of naturally occurring CD4^+^ Treg cells constitutively expressing HLA-G (HLA-G^+^ CD4^+^ Treg cells), which have been identified in the peripheral blood of healthy donors and patients [15, 60, 61], was analyzed. The proportion of CD4^+^ T cells in the peripheral blood of leukemic patients and of healthy donors was similar (29.6 ± 18.59%, mean ± SD, *n* = 21 versus 31.43 ± 8.57%, mean ± SD, *n* = 16; Figure 3(a)). Interestingly, as for DC-10, the percentage of HLA-G^+^ CD4^+^ Treg cells was significantly higher in patients with HLA-G^+^ blasts than in those with HLA-G^−^ blasts (4.2 ± 5.79%, mean ± SD, *n* = 14 versus 0.44 ± 1.01%, mean ± SD, *n* = 6; *P* = 0.0072 Figures 3(b) and 3(c)). This is the first report demonstrating the presence of HLA-G-expressing DC-10 and CD4^+^ T cells in the peripheral blood of leukemic patients. Our findings indicate that the frequency of regulatory cells, DC-10 and HLA-G^+^ CD4^+^ T cells, is increased in patients with HLA-G-expressing blasts, supporting the hypothesis that the expression of HLA-G on blasts may be a strategy by which leukemia promotes a tolerogenic microenvironment limiting anti-tumor responses. This mechanism of immune escape has been previously proposed for solid tumor where both infiltrating cells and tumor cells can express HLA-G [16]. It remains to be defined whether HLA-G-expressing tolerogenic cells are present in the bone marrow of AML patients where leukemic blasts reside and proliferate before emerging in the periphery. Moreover, correlation studies between HLA-G expression on blasts and the frequency of DC-10 and HLA-G^+^ CD4^+^ T cells will elucidate whether the microenvironment enriched in immunomodulatory factors allows the recruitment or the induction of tolerogenic cells inhibiting the antileukemic effects.

**Figure 4:**
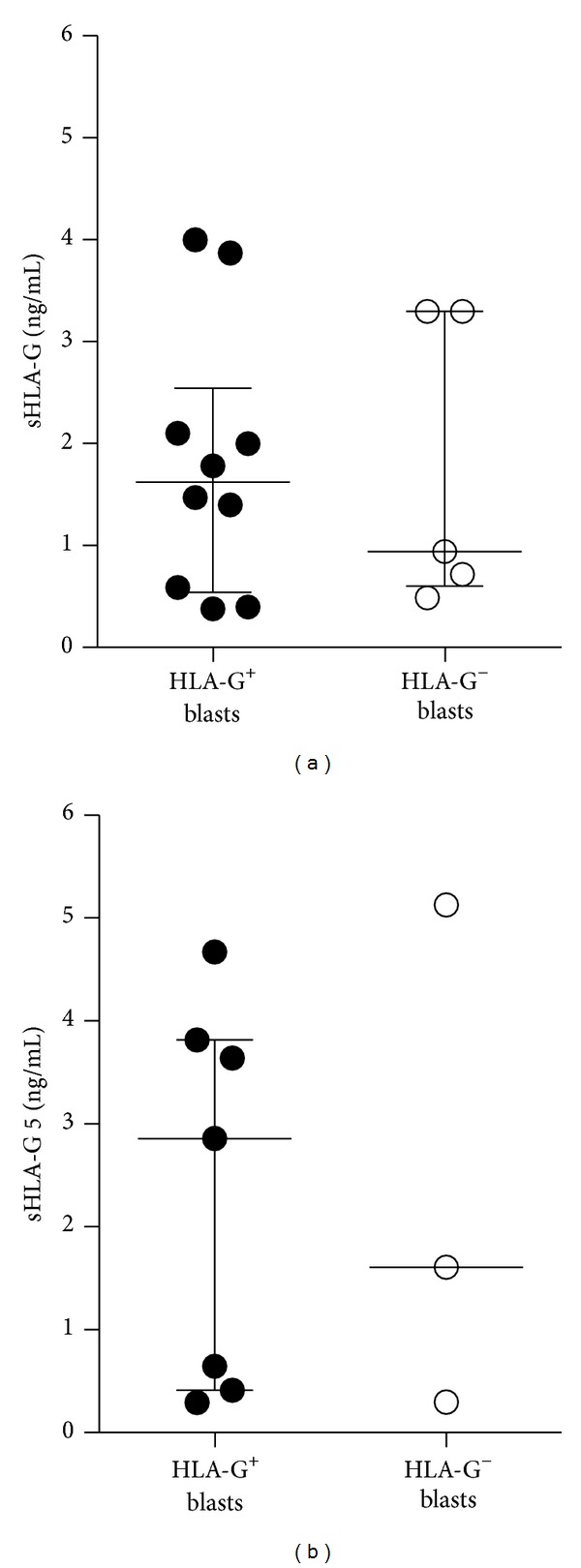
Soluble HLA-G levels in the sera of AML patients. The concentration of (a) sHLA-G (shed HLA-G1 and HLA-G5) and (b) HLA-G5 in the sera of AML patients was evaluated using specific sandwich ELISA. Each dot represents single AML patient. Lines indicate the median of serum levels (ng/mL) of positive samples in patients with HLA-G^+^ or HLA-G^−^ blasts.

Thus far, the presence of increased levels of soluble HLA-G has been associated with malignancies, including AML [25, 26]. We therefore sought to evaluate the amounts of soluble HLA-G in the serum of AML patients that were previously analyzed for HLA-G expression. sHLA-G (shed HLA-G1 and HLA-G5) was detected in 15 out of 18 patients with a mean value of positive samples of 1.78 ± 1.28 ng/mL (mean ± SD) and no differences were obtained between sera from patients with HLA-G^+^ and HLA-G^−^ blasts (1.79 ± 1.29 ng/mL, *n* = 10 versus 1.75 ± 1.42 ng/mL, *n* = 5, mean ± SD, Figure 4). In line with previous findings demonstrating that sHLA-G plasma levels were significantly higher in AML-M4 and AML-M5 acute leukemia subtypes [25], we detected higher amounts of sHLA-G in sera of AML-M4 patients as compared to those of other AML subtypes (2.8 ± 1.28 ng/mL *n* = 5 versus 1.2 ± 0.93 ng/mL, *n* = 10 mean ± SD; data not shown). In parallel, we detected HLA-G5 in 10 out of 18 patients with a mean value of positive samples of 2.33 ± 1.84 ng/mL (mean ± SD), and similar to sHLA-G, no differences were found between sera from patients with HLA-G^+^ and HLA-G^−^ blasts (2.33 ± 1.84 ng/mL, *n* = 7 versus 2.35 ± 2.5 ng/mL, *n* = 3; mean ± SD; Figure 4). Overall the levels of sHLA-G observed in our cohort of AML patients were lower compared to a previous work [25]. This discrepancy can be due to the fact that in our study we measured sHLA-G serum levels whereas Gros et al. [25] reported results from plasma samples. It has been indeed demonstrated that the levels of sHLA-G (shed HLA-G1 and HLA-G5) are significantly higher in plasma treated with EDTA as compared to those in plasma treated with heparin or in serum [62].

To define whether variations in the 3′UTR of HLA-G are associated with HLA-G expression, we analyzed 8 polymorphic sites at the* HLA-G* 3′UTR segment, including the 14 bp Ins/Del (rs1704), +3003 C/T (rs1707), +3010 C/G (rs1710), +3027 A/C (rs17179101), +3035 C/T (rs17179108), +3142 C/G (rs1063320), +3187 A/G (rs9380142), and +3196 C/G (rs1610696), previously described [34, 35]. The frequency of the 14 bp genotypes was similar in AML patients and healthy donors (Table 3). Since the 14 bp Ins is in strong linkage disequilibrium with the G in position +3142, we classified patients and controls according to the presence of the 14 bp Ins/Del and the +3142 C/G polymorphisms as InsG/InsG, DelC/DelC, DelC/InsG, and DelG/X. The relative frequencies of these genotypes in healthy donors were for InsG/InsG 23%, for DelC/DelC 22%, for DelC/InsG 32%, and for DelG/X 23% (Table 3). Interestingly, in AML patients we found a higher frequency of DelG/X genotype as compared to that observed in healthy donors (32% and 23%, resp.; Table 3). In line with these results, the frequency of UTR-3 haplotype (14 bp Del, +3003T, +3010C, +3027C, +3035C, +3142G, +3187A, and +3196C) was highly represented in AML patients than in healthy donors (16% and 9%, resp.; Table 3). Polymorphisms at the 3′UTR of* HLA-G* locus, and particularly the 14 bp Ins/Del, have been associated with different clinical conditions including autoimmunity and pathological pregnancy. So far, limited information has been published on the association of HLA-G polymorphisms in tumor cells with the levels of HLA-G expression and/or clinical outcome of patients [17]. Recently, studies in small cohort of patients investigated the association of 14 bp Ins/Del with the susceptibility to cervicovaginal and breast cancer, with controversial results [52, 63, 64]. Although in the present study the variation in the 3′UTR of HLA-G was evaluated in a limited number of AML patients, results showed no specific association, with the exception of UTR-3 haplotype. A more extensive study is warranted in a large cohort of patients in order to define whether specific UTRs of HLA-G might be associated with AML or can be used as genetic risk factor for the disease susceptibility.

## 4. Conclusions

Results from this study further improve the knowledge on the role of HLA-G in promoting tolerance. Moreover, they opem new clinical perspectives: HLA-G expression can be used as prognostic tumor biomarker to monitor disease state or as therapeutic target for improving immune responses against leukemia. The expression of HLA-G on blasts and the analysis of DC-10 and HLA-G^+^ CD4^+^ Tregs can be used to evaluate the effectiveness of anti-tumor therapies. Moreover, the analysis of HLA-G polymorphisms will allow the identification of specific HLA-G genotypes that could be associated with AML susceptibility.

## Figures and Tables

**Table 1:** Clinical patients' characteristics.

Variable	All patients	Blasts in PB Mean % (range)	HLA-G^+^ blasts *n* (mean %)	HLA-G^−^ blasts *n* (mean %)	*P* value^$^
Number of patients (%)	22	65.6 (13.3–96.4)	15 (68.2)	7 (31.8)	
Age at diagnosis (year)	59 (22–83)				
Male (%)	8 (36)	73.9 (13.3–94.5)	8 (100)	0 (0)	0.022
Female (%)	14 (64)	60.8 (23.9–96.4)	7 (50)	7 (50)	
AML	3	58.1 (42.2–85.2)	2 (66.7)	1 (33.3)	
AML-M0	2	35.6 (25.4–45.7)	1 (50)	1 (50)	
AML-M1	1	68	/	1 (100)	
AML-M2	7	62.4 (23.9–94.5)	5 (71.5)	2 (28.5)	
AML-M3	1	94.4	1 (100)	/	
AML-M4	6	81.5 (40–95.3)	4 (66.6)	2 (33.4)	
AML-M5a	1	96.4	1 (100)	/	
AML-M6	1	13.3	1 (100)	/	

^$^Comparison between HLA-G^+^ and HLA-G^−^ blasts using Fisher's exact test.

**Table 2:** HLA-G expression and cytogenetic karyotype.

Patient number	FAB classification	Sex	Age	HLA-G expression	Karyotype
1	AML-M2	F	83	Positive	46XX +8[4];[16]
3	sAML	F	69	Negative	46XX
5	AML-M4	F	54	Positive	46XX
9	AML-M4	F	44	Negative	46XX
10	AML-M2	M	60	Positive	46XY t(8; 21)(q22;q22), del(9)(q?22)
11	AML-M4	M	70	Positive	46XY[19]; +21[2]; iso p21[1]
18	AML-M2	F	68	Negative	46XX iso 8p
19	AML-M4	F	76	Positive	46XX
20	AML-M0	F	83	Positive	46XX −7/t(1;7;4;12)[12]
21	AML-M2	F	66	Positive	46XX +8[4];[16]
23	AML-M2	M	74	Positive	46XY
34	AML-M0	F	72	Negative	46XX
36	AML-M6	M	53	Positive	46XY complex(del5q,-10 14; 19, 7p-)[20]
37	AML-M2	F	59	Negative	46XX
38	AML	M	59	Positive	46XY
39	AML-M1	F	47	Negative	46XX
41	AML-M4	F	41	Negative	46XY
44	AML	M	36	Positive	46XY
45	AML-M4	F	44	Positive	46XX
49	AML-M2	M	77	Positive	46XY
54	AML-M3	M	22	Positive	46XY[6], t(15;17)[5]
55	AML-M5a	F	58	Positive	46XX

**Table 3:** Frequency of 3′UTR haplotypes and genotypes.

3′UTR polymorphic sites	AML patients (n = 19)	HDs (n = 141)
Ins/Ins	0.26	0.22
Del/Del	0.37	0.30
Ins/Del	0.37	0.47
InsG/InsG	0.26	0.23
DelC/DelC	0.15	0.22
InsG/DelC	0.26	0.32
DelG/X	0.32	0.23
UTR-1	0.21	0.32
UTR-2	0.29	0.3
UTR-3	0.16	0.09
UTR-4	0.13	0.11
UTR-5	0.03	0.05
UTR-6	0.05	0.02
UTR-7	0.08	0.08
UTR-8	0.05	0.032
